# 278. Immunocompromised Patients with Prolonged Viral Shedding of SARS-COV-2

**DOI:** 10.1093/ofid/ofab466.480

**Published:** 2021-12-04

**Authors:** Jessica Tarabay, Ahmed Babiker, Max W Adelman, Victoria D Stittleburg, Jay Varkey, Stephanie M Pouch, Jesse Waggoner, Anne Piantadosi

**Affiliations:** 1 Emory Healthcare, Atlanta, Georgia; 2 Emory University School of Medicine, Atlanta, GA; 3 Emory University, Atlanta, Georgia

## Abstract

**Background:**

Most individuals diagnosed with mild to moderate COVID-19 are no longer infectious after day 10 of symptom onset and those with severe or critical illness from COVID are typically not infection after day 20 day of symptom onset. Recovered persons can continue to test positive for SARS-CoV-2 by PCR via detection of non-viable RNA in nasopharyngeal specimens for up to three months (or longer) after illness onset. It is also know known that severely immunocompromised patients may produce replication-competent virus greater than 20 days from symptom onset and may require, per CDC recommendations, “additional testing and consultation with infectious diseases specialists and infection control experts”. We aim to discuss four case studies of severely immunocompromised patients who exhibited signs of persistent COVID-19 infection of COVID and how we managed transmission-based precautions in our hospital through sequencing and evaluation of cycle thresholds (CT) values and subgenomic RNA detection.

**Methods:**

Residual nasopharyngeal (NP) samples were collected on patients exhibiting persistent COVID like symptoms. These samples underwent N gene and N gene subgenomic RNA (sgRNA) real-time reverse transcription polymerase chain reaction (rRT-PCR) testing.

**Results:**

Analysis of longitudinal SARS-CoV-2 sequence data demonstrated within-patient virus evolution, including mutations in the receptor binding domain and deletions in the N-terminal domain of the spike protein, which have been implicated in antibody escape. See Figures 1 and 2.

Figure 1. Timelines of Identified Patients 1 and 2

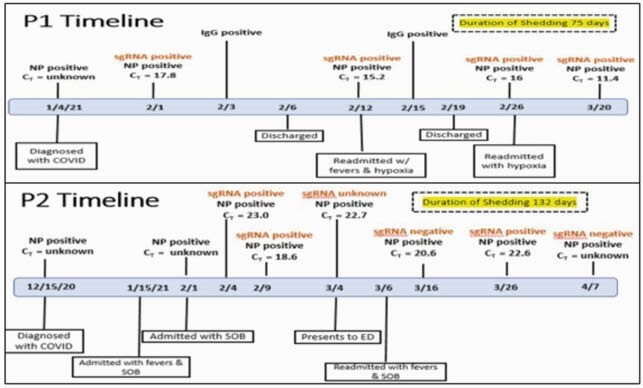

Patient 1: 46-year-old woman with recently diagnosed stage IV diffuse large B-cell lymphoma for which she was treated with 2 cycles of R-CHOP. Patient 2: 38-year-old woman with history of myelodysplastic syndrome, peripheral blood stem cell transplant with chronic graft versus host disease of the GI tract, skin, and eyes as well as CMV enteritis, and she was maintained on rituximab, mycophenolate mofetil, prednisone, and monthly IVIG without recent changes to her immunosuppression.

Figure 2. Timeline of Identified Patients 3 and 4

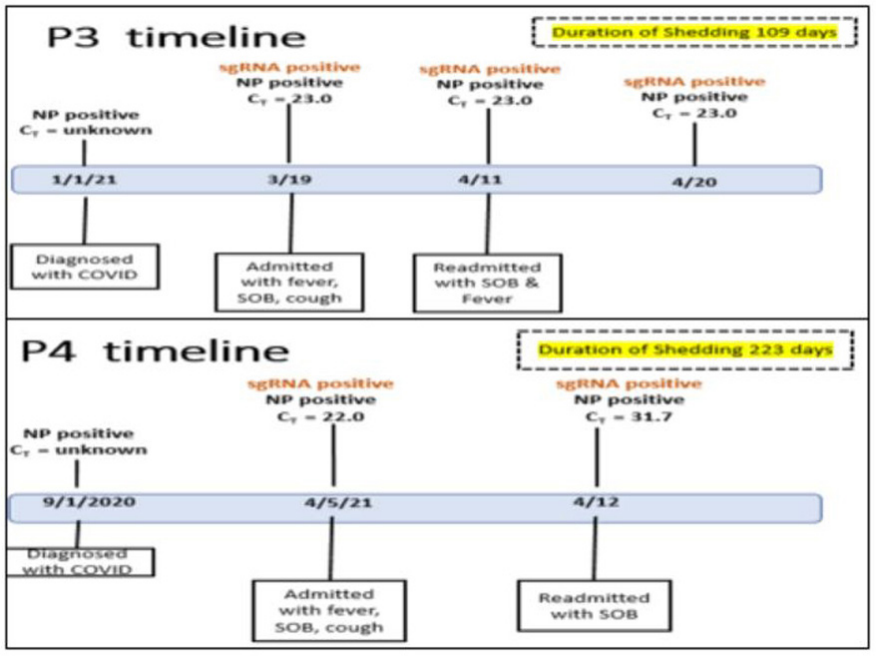

Patient 3: 44 year-old man with prior history of thymoma s/p thymectomy Patient 4: 46 year-old man who was initially diagnosed with marginal zone lymphoma approximately 2.5 years ago. He was initially treated with bendamustine and rituximab and achieved remission. He was then continued on maintenance rituximab without significant complications for a planned two years.

**Conclusion:**

Differentiating between prolonged viral shedding of non-infectious RNA and persistent replicating viable virus can be difficult to determine without full evaluation of a patient’s clinical picture and timeline. Consultation between laboratory, infectious diseases, and infection prevention experts to provide appropriate level of guidance for precautions and treatment may be warranted. Testing by PCR and analysis of CT values may provide key findings of viral replication in immunocompromised hosts, indicating the need for evaluation of additional treatment and maintaining isolation status in healthcare settings.

**Disclosures:**

**All Authors**: No reported disclosures

